# Adaptation and validation of the Swedish Five Factor Obsessive-Compulsive Inventory-Short Form in adults with and without eating disorder psychopathology

**DOI:** 10.1186/s12888-026-08355-9

**Published:** 2026-07-15

**Authors:** Ivan Ejdemyr, Miguel Inzunza, Kirsten Gilbert, Johanna Levallius, Magnus Sjögren

**Affiliations:** 1 Department of Clinical Sciences, Umeå University, Umeå, Sweden; 2 National Specialized Medical Care for Eating Disorders, Region Västernorrland, Sundsvall, Sweden; 3 Unit of Police Work/Research Unit, Umeå University, Umeå, Sweden; 4 Department of Psychiatry, Washington University School of Medicine, St. Louis, MO USA

**Keywords:** Five Factor Obsessive-Compulsive Inventory-Short Form, Obsessive-compulsive personality traits, Psychometric validation, Eating disorders, Maladaptive personality traits, Five-factor model of personality

## Abstract

**Background:**

Obsessive–compulsive personality disorder (OCPD) traits are highly prevalent in eating disorder populations and linked to greater symptom severity and poorer treatment outcomes, yet no validated dimensional measure of these traits exists for use in Swedish-speaking populations or in eating disorder contexts. The Five-Factor Obsessive–Compulsive Inventory–Short Form (FFOCI-SF) is a brief, dimensional instrument designed to assess OCPD-related traits within the five-factor model framework. This study aimed to adapt and validate the Swedish FFOCI-SF and to examine its psychometric functioning in adults with and without elevated eating disorder symptoms.

**Methods:**

Adults (*n* = 395) completed the FFOCI-SF, NEO-PI-3, DIP-Q, and EDE-Q. Psychometric properties were examined using a multimethod approach integrating structural equation modeling and classical test theory.

**Results:**

A revised 10-factor, 41-item model, reflecting the merger of three facets (C1–C3), demonstrated improved fit, clearer factor delineation, and acceptable-to-excellent internal consistencies (α = 0.66–0.93). Convergent and discriminant validity were supported, and facet intercorrelations broadly aligned with theoretical expectations. Full measurement invariance was not supported; however, reliability, facet intercorrelations, and associations with external criteria were broadly similar across groups. Preliminary group comparisons suggested higher scores across most facets among participants with elevated eating disorder symptoms.

**Conclusions:**

The revised Swedish FFOCI-SF demonstrates promising psychometric properties and a clinically interpretable 10-facet structure as a dimensional measure of OCPD traits. The findings further provide initial evidence for its applicability in eating disorder samples.

**Supplementary Information:**

The online version contains supplementary material available at 10.1186/s12888-026-08355-9.

## Background

Obsessive–compulsive personality disorder (OCPD) has been included in the *Diagnostic and Statistical Manual of Mental Disorders* since its first edition, underscoring its long-standing clinical relevance [17]. In the current edition, OCPD is defined by rigidity, perfectionism, excessive devotion to productivity, and preoccupation with rules, control, and order [1]. Within dimensional models of personality pathology, OCPD is primarily conceptualized as a maladaptive extension of high conscientiousness [35]. This dimensional perspective is increasingly recognized for its clinical and empirical advantages [21]. OCPD is the most common personality disorder [10] and is associated with marked functional impairment [28, 31], elevated clinical risk [5], and high comorbidity with psychiatric conditions, including eating disorders [12]. In eating disorder populations, OCPD traits are frequently observed even in the absence of a formal OCPD diagnosis and are linked to greater symptom severity and poorer treatment outcomes [15, 39]. This highlights the need for a psychometrically robust, dimensional instrument for accurate assessment and intervention.

One instrument developed for this purpose is the Five-Factor Obsessive–Compulsive Inventory–Short Form (FFOCI-SF; [18]), a brief version of the original FFOCI [36]. The FFOCI-SF assesses 12 maladaptive personality facets that operationalize OCPD within the five-factor model of personality [26]. These facets were derived through expert ratings, systematic reviews of historical and contemporary definitions of OCPD, and empirical trait research. Although they primarily reflect maladaptive variants of conscientiousness (e.g., perfectionism, workaholism), the facets also capture theoretically relevant variance associated with neuroticism, extraversion, and openness [36]. Importantly, the FFOCI framework was designed to assess a multidimensional profile of OCPD-relevant traits rather than an OCPD syndrome or a single higher-order factor [36]. To enhance efficiency and clinical utility, the 48-item FFOCI-SF was developed by selecting the strongest-performing items from the full instrument using confirmatory factor analysis (CFA) and item-response theory, to preserve the intended facet structure and nomological network of the original FFOCI [18]. However, findings from a language adaptation of the FFOCI-SF suggest that several conscientiousness-related facets may be difficult to distinguish empirically using CFA [24], underscoring the need to systematically evaluate the replicability and dimensional distinctiveness of the proposed factor structure across languages and clinical contexts.

### The current study

To date, no validated Swedish dimensional measure of OCPD traits is available, limiting both research and clinical assessment. Neither the FFOCI nor the FFOCI-SF has been evaluated in Swedish populations, and neither version has been examined in eating disorder populations, where OCPD traits are highly prevalent and clinically important. Given the practicality and intended clinical utility of the FFOCI-SF, the current study aimed to adapt and validate the instrument for use in a Swedish adult population, including both an eating disorder group and a non-clinical comparison group. Specifically, the aims were to: (a) evaluate item-level properties and internal consistency; (b) test the hypothesized 12-factor structure and assess whether empirical refinements were warranted; (c) examine the stability of the factor structure across the eating disorder and non-clinical groups, including a stepwise assessment of measurement invariance; (d) compare group differences in FFOCI-SF scores at both the latent and observed-score levels, informed by the invariance results; (e) examine intercorrelations among the facets; and (f) evaluate convergent and discriminant validity in relation to established measures of normal-range personality traits and OCPD.

## Methods

### Participants and procedure

Recruitment was conducted in Sweden, and data collection was conducted online between March and August 2024. Participants were recruited through four channels: (1) a dedicated study website, (2) targeted social media advertisements and posts, (3) printed flyers at Umeå University, and (4) flyers distributed at outpatient psychiatric clinics. Recruitment materials aimed to reach both the general population and individuals with elevated eating disorder symptoms. On social media, five paid advertisement campaigns were run through the study’s Facebook page, targeting demographic and lifestyle factors previously associated with eating disorders (e.g., women, exercise, vegan diet). Additional posts were made in two eating disorder–specific Facebook forums and several student forums to reach a broader population.

Across channels, participants accessed the same study website and were presented with standardized study information. Informed consent was given by selecting a checkbox. After consenting, participants completed a fixed questionnaire battery in the following order: (1) EDE-Q, (2) demographic questions, (3) FFOCI-SF, (4) NEO-PI-3, and (5) DIP-Q (all instruments are described under Measures). The battery could be completed at the participant’s own pace and was estimated to take approximately 45 min. Participants were informed that they could withdraw at any time or pause and return later using a unique REDCap access code. REDCap prompted participants to respond to each item before advancing. Upon completion, participants received 100 SEK (approximately $10 USD).

Inclusion criteria were age ≥ 18 years and provision of informed consent. Participants were excluded if they provided inconsistent responses to any of five embedded attention check items (see Measures), used as an indirect indicator of inattentive responding or insufficient Swedish comprehension. A total of 438 individuals completed the questionnaire battery; 42 (9.6%) were excluded for one or more inattentive responses, and one duplicate entry was removed, resulting in a final sample of 395 participants. To ensure variability in eating disorder symptomatology, participants were stratified based on their EDE-Q scores. Consistent with established Swedish cut-offs [42], scores ≥ 2.83 indicated clinically significant symptomatology. Participants scoring ≥ 2.83 are referred to as the eating disorder group and those scoring < 2.83 as the non-clinical comparison group, although this grouping does not imply verified eating disorder diagnoses. Using this classification, 152 participants were allocated to the eating disorder group and 243 to the non-clinical comparison group. Descriptive statistics for the full sample and both groups are presented in Table 1.

**Table 1:** Demographic and clinical characteristics of the total, eating disorder, and non-clinical samples

	Total sample	Non-clinical sample	Eating disorder sample
	(*n* = 395)	(*n* = 243)	(*n* = 152)
Age, years, mean (SD)	35.79 (11.81)	34.93 (11.39)	37.16 (12.38)
Gender, %, (*n*)			
Women	87.8% (347)	85.6% (208)	91.4% (139)
Men	10.4% (41)	12.8% (31)	6.6% (10)
Other	1.8% (7)	1.6% (4)	2% (3)
Occupational status, %, (*n*)			
Employed	47.3% (187)	48.6% (118)	45.4% (69)
On sick leave	10.9% (43)	5.3% (13)	19.7% (30)^*^
Unemployed	3.5% (14)	4.5% (11)	2% (3)
Student	32.9% (130)	38.3% (93)	24.3% (37)^*^
Other	5.3% (21)	3.3% (8)	8.6% (13)^*^
Highest level of education, %, (*n*)			
Incomplete secondary	5.6% (22)	3.3% (8)	9.2% (14)^*^
Completed secondary	19.2% (76)	16.5% (40)	23.7% (36)
< 3 years of university	18.2% (72)	17.3% (42)	19.7% (30)
≥ 3 years of university	57% (225)	63% (153)	47.4% (72)^*^
Psychiatric diagnosis†, %, (*n*)			
Any psychiatric diagnosis	48.1% (190)	34.6% (84)	69.7% (106)^*^
ADHD	10.4% (41)	5.8% (14)	17.8% (27)^*^
Anorexia nervosa	12.4% (49)	2.9% (7)	27.6% (42)^*^
ASD	4.8% (19)	2.9% (7)	7.9% (12)^*^
Bipolar disorder	3.8% (15)	2.5% (6)	7.2% (9)
Binge-eating disorder	1.5% (6)	1.3% (3)	2% (3)
Bulimia nervosa	2.5% (10)	1.6% (4)	3.9% (6)
GAD	9.9% (39)	8.2% (20)	12.5% (19)
Major depressive disorder	24.1% (95)	17.3% (42)	34.9% (53)^*^
Obsessive-compulsive disorder	3.3% (13)	1.6% (4)	5.9% (9)^*^
PTSD	9.4% (37)	6.2% (15)	14.5% (22)^*^
EDE-Q, mean (SD)	2.33 (1.63)	1.1 (0.76)	3.9 (0.79)^*^

Note. ADHD = attention-deficit/hyperactivity disorder; ASD = autism spectrum disorder; GAD = generalized anxiety disorder; PTSD = post-traumatic stress disorder; ED = eating disorder; EDE-Q = Eating Disorder Examination Questionnaire; *n* = number; SD = standard deviation. †Participants could endorse more than one diagnosis. *p* < .05 (*)

### Measures

#### Five-Factor Obsessive-Compulsive Inventory-Short Form (FFOCI-SF)

The FFOCI-SF is the 48-item short form of the FFOCI [36], designed to assess 12 maladaptive personality facets associated with OCPD, as conceptualized within the five-factor model [18]. The 12 facets are: Perfectionism (C1), Fastidiousness (C2), Punctiliousness (C3), Workaholism (C4), Doggedness (C5), Ruminative Deliberation (C6), Detached Coldness (E1), Risk Aversion (E5), Excessive Worry (N1), Constricted (O3), Inflexibility (O4), and Dogmatism (O6). Each facet is measured by four items rated on a 5-point Likert scale from 1 (“not at all true”) to 5 (“completely true”), with higher scores indicating greater maladaptive trait expression. Across the full instrument, three items are reverse-scored.

For this study, the FFOCI-SF was translated into Swedish in accordance with World Health Organization guidelines [43]. The first author (IE), a native Swedish speaker fluent in English, conducted the initial translation. Two co-authors (JL, MS) reviewed and refined the wording until consensus was reached. A bilingual Swedish-speaking PhD residing in the United States, blind to the original instrument, carried out a back-translation. This version was reviewed by the original instrument developer (Douglas Samuel), and any discrepancies were resolved through discussion. The preliminary Swedish version was piloted in a small sample to assess clarity and comprehension, and minor adjustments were made before the final version was established. For transparency, Supplementary Table 1 provides the original English items, Swedish translations, original facet assignments, final revised facet assignments, and retained/removed status for all items. The original 48-item and revised 41-item scoring algorithms are provided in Supplementary Tables 2 and 3, respectively.

#### NEO Personality Inventory-3 (NEO-PI-3)

The NEO-PI-3 is a 240-item self-report inventory assessing the five major domains of the five-factor model and their 30 lower-order facets [26]. Items are rated on a 5-point Likert scale ranging from 0 (“not at all true”) to 4 (“completely true”). The Swedish version has demonstrated satisfactory psychometric properties in all lower-order facets, except the Openness to Values (O6) facet [23], and the five-factor structure has been replicated in psychiatric samples [3]. Cronbach’s alpha in the current sample was generally satisfactory (α = 0.50–0.90), with lower alphas for Excitement Seeking (E5, α = 0.60) and Openness to Values (O6, α = 0.50).

#### DSM-IV and ICD-10 Personality Questionnaire (DIP-Q)

The DIP-Q is a 140-item self-report questionnaire assessing all DSM-IV and ICD-10 personality disorders [30]. The OCPD/anankastic section consists of 12 items, originally forming two highly overlapping scales (DSM-IV and ICD-10). A prior validation reported internal consistency estimates of α = 0.55 and 0.64, respectively, and high correlations between the two scales (*r* = .87; [30]). In the present study, all 12 items were combined into a single OCPD scale (α = 0.52), consistent with the conceptual heterogeneity of OCPD and previous Swedish validations [4, 30]. Two items assessing hoarding and miserliness showed negative item–total correlations, which is consistent with prior evidence that these criteria function atypically and load weakly or not at all on core OCPD dimensions [16, 33]. These items were therefore excluded, yielding a 10-item scale with improved internal consistency (α = 0.64).

#### Eating Disorder Examination Questionnaire version 6.0 (EDE-Q)

The EDE-Q is a 28-item self-report instrument that assesses eating disorder psychopathology, with items rated on a 7-point Likert scale (0–6), where higher scores reflect greater severity [13]. The Swedish version has robust psychometric properties and well-established cut-off scores for both non-clinical and clinical eating disorder populations [42]. Cronbach’s alpha in the current sample was 0.96.

#### Demographic questionnaire

Participants reported age, sex, education, employment status, and formal psychiatric diagnoses received from a licensed healthcare provider within the past 24 months. Diagnoses were self-reported and not verified against medical records.

#### Attention check items

Five embedded attention check items were included within the FFOCI-SF and NEO-PI-3 (together ~ 90% of all items) and were rated on the same 5-point Likert scale. Items consisted of implausible or trivially true statements (e.g., “*I have been to the moon*”; “*Stockholm is the capital of Sweden*”), written in both positive and negative directions to detect patterned or inattentive responding.

### Statistical analysis

Following recommendations for evaluating multidimensional personality instruments [20], this study employed a multimethod psychometric approach that integrated structural equation modeling (SEM) and classical test theory (CTT). All analyses were conducted using IBM SPSS Statistics (version 26) and Mplus version 8.10 [29]. Analyses were performed in the total sample and, where relevant, separately within the eating disorder and non-clinical comparison groups. Statistical significance was defined as *p* < .05 (two-tailed).

The internal structure of the hypothesized 12-factor model was evaluated using confirmatory factor analysis (CFA), consistent with prior work establishing this structure [18, 36]. Multiple fit indices and statistical tests were employed to assess fit of models [6, 7, 22]. The chi-square statistic and its degrees of freedom were reported for completeness, acknowledging that the test is highly sensitive to sample size and therefore tends to reject even reasonably well-fitting models in larger samples. Consequently, greater emphasis was placed on approximate fit indices, including the root mean square error of approximation (RMSEA) and its 90% confidence interval. For RMSEA, cut-off values below 0.08 were used to indicate acceptable fit. Additionally, the comparative fit index (CFI) was applied. We also included the gamma hat index, which is considered more stable and interpreted similarly to the CFI [14]. Finally, the standardized root mean square residual (SRMR) was assessed, using a cutoff value of 0.08. To account for deviations from normality, the models were estimated with robust maximum likelihood (MLR). Measurement invariance across groups was examined using a stepwise multigroup CFA approach, testing configural, metric, and scalar invariance, in line with recommended procedures for comparing latent variables [6, 8]. Invariance decisions were based on changes in model fit, including non-significant chi-square difference tests and ΔCFI ≤ 0.01 [9]. Latent group differences were examined within the multigroup CFA framework where supported by the invariance results. Observed-score group differences in FFOCI-SF facets were tested using independent-samples t-tests.

Item- and scale-level properties were evaluated within a CTT framework. For each item, descriptive statistics (mean, standard deviation, skewness, kurtosis), corrected item–total correlations (CITC), Cronbach’s alpha if item deleted, and interitem correlations were computed. Internal consistency (Cronbach’s alpha) was estimated for each subscale in the full sample and separately within the eating disorder and non-clinical groups. Intercorrelations among the facet scales were calculated using Pearson’s r. SEM-based latent correlations with the NEO-PI-3 and the DIP-Q were treated as the primary analyses of convergent validity. Convergent validity with the NEO-PI-3 was evaluated against theoretically corresponding five-factor facets: FFOCI-SF Excessive Worry (N1) with NEO-PI-3 Anxiety (N1); Detached Coldness (E1) with Warmth (E1; negative direction); Risk Aversion (E5) with Excitement Seeking (E5; negative direction); Constricted (O3) with Feelings (O3; negative direction); Inflexibility (O4) with Actions (O4; negative direction); Dogmatism (O6) with Values (O6; negative direction); Workaholism (C4) with Achievement Striving (C4); Doggedness (C5) with Self-Discipline (C5); and Ruminative Deliberation (C6) with Deliberation (C6). For the revised rigid conscientious control factor (C1–C3), derived from the original Perfectionism (C1), Fastidiousness (C2), and Punctiliousness (C3) facets, convergent validity was evaluated against NEO-PI-3 Competence (C1), Order (C2), and Dutifulness (C3). Because the DIP-Q OCPD scale reflects traditional DSM/ICD-based OCPD criteria, we expected stronger associations with facets indexing perfectionistic, rigid, rule-bound, and maladaptive conscientiousness-related content, and weaker associations with facets indexing interpersonal-affective restriction, particularly Detached Coldness (E1) and Constricted (O3). Discriminant validity, and complementary analyses of convergent validity, were examined using observed-score correlations. Discriminant indices were summarized as the mean (range) correlation with non-corresponding facets within and outside the domain. Group differences in demographic variables were examined using independent-samples t-tests and chi-square tests. Effect sizes were expressed as Cohen’s d [11]. Differences in observed correlation strength between the groups were tested with Fisher’s r-to-z transformation.

### Data management and ethical considerations

Data were collected and managed using REDCap [19] and stored on secure university servers in accordance with Swedish data protection regulations. The study was approved by the Swedish Ethical Review Authority (reference number 2023-02949-02). All participants provided informed consent before participation, and data were fully anonymized.

## Results

### Sample characteristics

Descriptive characteristics for the total sample (*n* = 395) and the subsamples (eating disorder group, *n* = 152; non-clinical group, *n* = 243) are presented in Table 1. As expected given stratification, the eating disorder group showed markedly higher eating disorder symptomatology scores. Compared to the non-clinical comparison group, the eating disorder group was more often on sick leave, less often students, and had lower rates of higher education. The eating disorder group also reported higher prevalence of psychiatric diagnoses, including anorexia nervosa, obsessive-compulsive disorder, and major depressive disorder. Unless otherwise specified, the following analyses were conducted in the full sample.

### Item- and scale-level psychometrics

#### SEM results: Evaluation of the 12-factor model

**Fig. 1:**
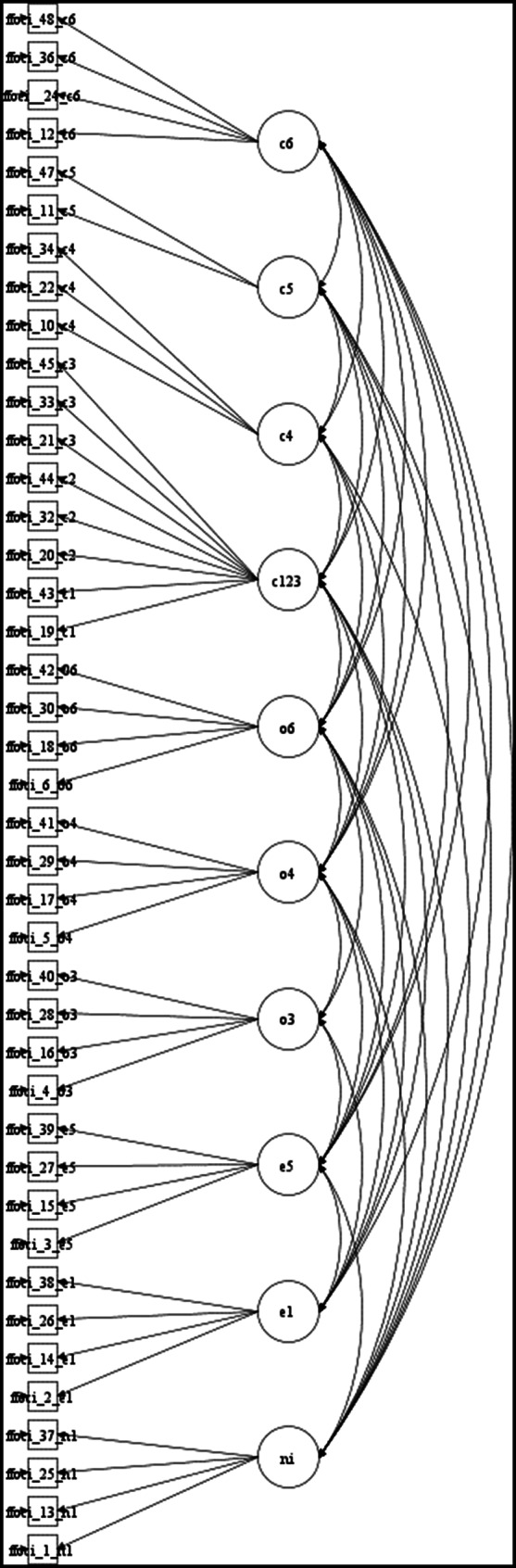
Correlated 10-factor, 41-item measurement model of the revised FFOCI-SF

The initial CFA of the 48-item, 12-factor model was conducted in the full sample (*n* = 395) and yielded the following fit indices: χ²(1014) = 2477.523, *p* < .01, CFI = 0.82, gamma hat index = 0.87, RMSEA = 0.060, 90% CI [0.057, 0.063], and SRMR = 0.08. Although several indices indicated acceptable fit, the CFI and gamma hat index fell below recommended thresholds (CFI = 0.82; gamma hat = 0.87), suggesting suboptimal model fit and motivating further model refinement. Inspection of the solution revealed items with weak factor loadings, substantial cross-loadings, and several highly correlated factors, indicating empirical overlap among facets. Model respecification therefore focused on the removal of weak or cross-loading items and the consolidation of empirically redundant factors. Following these adjustments, a revised 41-item, 10-factor model was retained, yielding improved fit indices: χ²(734) = 1528.175, *p* < .01, CFI = 0.89, gamma hat index = 0.92, RMSEA = 0.052, 90% CI [0.049, 0.056], and SRMR = 0.06. Most indices indicated acceptable fit; although the CFI remained slightly below conventional cutoffs (0.89), the gamma hat index suggested good fit (0.92). Standardized factor loadings ranged from 0.387 to 0.938. The main structural change from the initial model was the consolidation of C1, C2, and C3 into a single factor (Fig. 1), hereafter referred to as C1–C3 and interpreted as rigid conscientious control. Moreover, seven items were removed due to weak primary loadings, substantial cross-loadings, or empirical redundancy within the conscientiousness-related facets. Items 7 and 31 (C1) primarily reflected weak loading patterns, item 8 (C2) showed an ambiguous loading pattern, items 9 (C3), 35 (C5), and 46 (C4) primarily reflected cross-loading patterns, and item 23 (C5) was removed because it was highly redundant with item 11. To support interpretation of these refinements, item wording, original and revised facet assignments, and retained/removed status are detailed in Supplementary Table 1. The original 48-item and revised 41-item scoring algorithms are shown in Supplementary Tables 2 and 3, respectively.

#### CTT results: Item properties and reliability

Since the revised SEM solution involved merging facets and removing items, we report both the item-level properties for the original 48-item, 12-facet structure and the scale-level properties for the revised 41-item, 10-facet solution. Full item-level statistics for the original 12-facet structure are presented in Supplementary Table 4. All items demonstrated full use of the 5-point response scale and adequate variability. Skewness and kurtosis values were within acceptable limits. CITCs showed substantial item-level heterogeneity, with most facets demonstrating a mix of moderate-to-strong items and a few weaker items. The only facet showing consistently poor coherence was the original C1 facet, which contained several low-CITC items and correspondingly low internal consistency (α = 0.54). Internal consistency was acceptable to excellent across facets in the revised 10-facet solution (α = 0.66–0.93), and mean interitem correlations were moderate on average, with variability consistent with differences in facet breadth (Supplementary Table 5). Cronbach’s alpha values were comparable across eating disorder and non-clinical samples (Δα ≈ 0.00–0.08). From this point forward, unless otherwise specified, all analyses refer to the revised 41-item, 10-factor model.

### Group differences: latent and observed mean differences

Measurement invariance analyses of the 10-factor model did not support configural, metric, or scalar invariance across the eating disorder and non-clinical groups, likely reflecting model complexity and unequal group sizes [6]. Furthermore, C5 could not be evaluated at the latent level because it contained only two items. Group comparisons were therefore interpreted as exploratory known-groups analyses. Latent mean comparisons indicated higher scores in the eating disorder group across all evaluable facets except O3, which showed no significant difference (Table 2). Observed-score comparisons yielded a highly similar pattern (Table 3), with statistically significant group differences for all revised facets except O3 and effect sizes ranging from small to large.

**Table 2:** Latent mean differences between eating disorder and non-clinical groups across FFOCI-SF facets

Facet	Non-clinical latent mean^a^
C1–C3†‡	–0.50^*^
C4†‡	–0.31^*^
C5†§	–––
C6‡	–0.47^*^
N1	–0.70^*^
E1‡	–0.14^*^
E5	–0.24^*^
O3	–0.08
O4	–0.27^*^
O6	–0.35^*^

Note. *N* = 395 (eating disorder = 152; non-clinical = 243); †Revised FFOCI-SF facets reflect the final 41-item configuration; C1–C3 = combined Perfectionism, Fastidiousness, and Punctiliousness, interpreted as rigid conscientious control; C4 = Workaholism; C5 = Doggedness; C6 = Ruminative Deliberation; N1 = Excessive Worry; E1 = Detached Coldness; E5 = Risk Aversion; O3 = Constricted; O4 = Inflexibility; O6 = Dogmatism; ^‡^Metric–scalar invariance (ΔCFI ≤ 0.01) was not supported for this factor; ^§^C5 could not be evaluated at the latent level because it contains only two items; ^a^Latent means for the eating disorder group were fixed to zero for model identification; values reflect non-clinical group means relative to the eating disorder group. Negative values therefore indicate lower scores in the non-clinical group, corresponding to higher scores in the eating disorder group; *p* < .05 (*)

**Table 3:** Observed-score mean differences between eating disorder and non-clinical groups across FFOCI-SF facets

	Eating disorder group	Non-clinical group	Cohen’s d	95% CI	
	(*n* = 152)	(*n* = 243)		LL	UL
C1–C3†	3.34 (0.76)	2.87 (0.75)^*^	0.62	0.31	0.62
C4†	2.89 (1.05)	2.52 (0.86)^*^	0.40	0.18	0.56
C5†	3.48 (0.93)	3.16 (0.97)^*^	0.34	0.12	0.52
C6	3.47 (1.02)	2.99 (0.95)^*^	0.49	0.28	0.68
N1	4.05 (0.90)	3.25 (1.15)^*^	0.75	0.58	1.01
E1	2.14 (0.82)	1.92 (0.74)^*^	0.28	0.06	0.37
E5	3.36 (0.77)	3.02 (0.78)^*^	0.44	0.18	0.50
O3	2.00 (0.79)	1.86 (0.72)	0.18	-0.01	0.29
O4	3.07 (0.84)	2.73 (0.79)^*^	0.41	0.16	0.49
O6	2.63 (0.80)	2.23 (0.69)^*^	0.55	0.25	0.55

*Note. N* = 395 (eating disorder = 152; non-clinical = 243). † Revised FFOCI-SF facets reflect the final 41-item, 10-factor configuration supported by the SEM analyses. C1–C3 = combined Perfectionism, Fastidiousness, and Punctiliousness, interpreted as rigid conscientious control; C4 = Workaholism; C5 = Doggedness; C6 = Ruminative Deliberation; N1 = Excessive Worry; E1 = Detached Coldness; E5 = Risk Aversion; O3 = Constricted; O4 = Inflexibility; O6 = Dogmatism. Values in the eating disorder and non-clinical columns represent means, with standard deviations in parentheses. Cohen’s d is calculated such that positive values indicate higher scores in the eating disorder group; *p* < .05 (*)

### Facet interrelationships

Using the revised 10-facet structure, intercorrelations ranged from negligible to large, with most associations falling within the small-to-moderate range (Table 4). The strongest associations were observed within the conscientiousness domain, where the merged C1–C3 facet correlated strongly with C4, C5, and C6 (rs = 0.56–0.63). O3 showed a distinct profile, with near-zero correlations with all conscientiousness facets and with N1 (rs ≈ –0.05 to 0.08), but modest correlations with the other openness facets (rs = 0.20–0.24). Its strongest association was with E1 (*r* = 0.62). E1 showed a parallel pattern of weak associations with conscientiousness facets (rs = –0.02 to 0.15), but moderate correlations with other domains. Exploratory Fisher’s r-to-z tests indicated highly similar correlation structures across eating disorder and non-clinical participants, with only one small difference (C6–N1; Supplementary Table 6).

**Table 4:** Observed intercorrelations among FFOCI-SF subscales

	C1-C3†	C4†	C5†	C6	E1	E5	N1	O3	O4	O6
C1-C3†		0.56^*^	0.61^*^	0.63^*^	0.13^*^	0.38^*^	0.36^*^	0.08	0.48^*^	0.52^*^
C4†			0.48^*^	0.22^*^	− 0.02	0.02	0.11^*^	0.04	0.16^*^	0.29^*^
C5†				0.40^*^	0.03	0.22^*^	0.21^*^	0.05	0.35^*^	0.38^*^
C6					0.15^*^	0.48^*^	0.52^*^	0.08	0.48^*^	0.40^*^
E1						0.23^*^	0.12^*^	0.62^*^	0.27^*^	0.20^*^
E5							0.44^*^	0.15^*^	0.74^*^	0.29^*^
N1								− 0.05	0.38^*^	0.24^*^
O3									0.24^*^	0.20^*^
O4										0.42^*^

Note. *n* = 395. Only the upper triangle is reported; cells below the diagonal are intentionally left blank to avoid redundancy. FFOCI-SF = Five-Factor Obsessive–Compulsive Inventory–Short Form (41 item -item revised version). †Post-SEM revised scales are based on the final item configuration. FFOCI-SF subscales: C1–C3† = combined Perfectionism, Fastidiousness, and Punctiliousness, interpreted as rigid conscientious control; C4† = Workaholism; C5† = Doggedness; C6 = Ruminative Deliberation; E1 = Detached Coldness; E5 = Risk Aversion; N1 = Excessive Worry; O3 = Constricted; O4 = Inflexibility; O6 = Dogmatism. *p* < .05 (*)

### Convergent and discriminant validity

Convergent validity was primarily evaluated using SEM-based latent correlations with external criteria (Table 5). Convergent validity with theoretically corresponding NEO-PI-3 facets was supported, with associations generally ranging from moderate to very strong in magnitude (|r| ≈ 0.50–0.97). Convergent validity with the DIP-Q OCPD was also supported, with associations ranging from small to large in magnitude (|r| ≈ 0.21–0.82), with comparatively weaker associations observed for E1 and O3, and the strongest associations observed for the combined C1–C3 facet and C6. Complementary CTT-based analyses showed that discriminant correlations with non-corresponding NEO facets were substantially lower than convergent correlations, averaging close to zero, and that the overall convergent pattern mirrored the SEM-based results (Supplementary Table 7). Exploratory Fisher’s r-to-z comparisons indicated no significant differences between the eating disorder sample and non-clinical sample (Supplementary Table 8).

**Table 5:** Latent convergent validity of the FFOCI-SF

	C1-C3†	C4†	C5†	C6	N1	E1	E5	O3	O4	O6
NEO facet^‡^	*n*/a§	0.78^*^	0.90^*^	0.72^*^	0.96^*^	− 0.88^*^	− 0.70^*^	*n*/a¶	− 0.97^*^	− 0.50^*^
DIP-Q OCPD	0.82^*^	0.55^*^	0.53^*^	0.73^*^	0.66^*^	0.26^*^	0.61^*^	0.21^*^	0.62^*^	0.54^*^

Note. *n* = 395. Values are standardized latent correlations estimated using structural equation modeling. †Revised FFOCI-SF scales reflect the final 41-item configuration. FFOCI-SF subscales: C1–C3† = combined Perfectionism, Fastidiousness, and Punctiliousness, interpreted as rigid conscientious control; C4† = Workaholism; C5† = Doggedness; C6 = Ruminative Deliberation; N1 = Excessive Worry; E1 = Detached Coldness; E5 = Risk Aversion; O3 = Constricted; O4 = Inflexibility; O6 = Dogmatism. DIP-Q OCPD = DSM-IV and ICD-10 Personality Questionnaire (10 OCPD items). NEO facet‡ = corresponding Revised NEO-PI-3 facet. §For C1–C3, convergent validity was evaluated against NEO C1 (0.04), NEO C2 (0.70*), and NEO C3 (0.49*). ¶Latent correlations involving NEO O3 could not be reliably estimated due to a non-positive definite latent covariance matrix, indicating model identification problems, and are therefore not reported. *p* < .05 (*)

## Discussion

This study evaluated the psychometric properties of the Swedish version of the FFOCI-SF in adults with and without elevated eating disorder symptoms. Overall, the original 12-factor structure received only partial support, whereas a revised 10-factor solution demonstrated improved model fit, acceptable to excellent reliability, and coherent validity patterns across measures. These refinements primarily involved the consolidation of overlapping conscientiousness facets (notably the merging of C1–C3) and targeted item removals, and psychometric functioning was broadly comparable across the eating disorder and non-clinical groups.

The merging of C1–C3 aligns with the only published CFA evaluation of the FFOCI-SF, which reported limited separability of several conscientiousness-related facets, including C1–C2 and C4–C5 [24]. Substantial overlap among these facets is also theoretically expected given their shared foundations in orderliness, self-regulation, dutifulness, and high conscientiousness [34]. In the present study, the revised C1–C3 factor is therefore interpreted as a broader facet of rigid conscientious control, reflecting perfectionistic standards, excessive order/detail orientation, and strict rule-bound responsibility. This interpretation preserves the theoretical link to the original FFOCI-SF facets while acknowledging that their intended distinctions were not empirically recoverable as separate short-form scales in this sample. The revised factor retained item content from all three original facets, including two Perfectionism (C1), three Fastidiousness (C2), and three Punctiliousness (C3) items. At the same time, its NEO-PI-3 convergent-validity profile was uneven, with stronger associations for Order (C2) and Dutifulness (C3) than for Competence (C1), which primarily reflects normal-range efficacy rather than maladaptive perfectionism per se. Taken together, the item content and validity pattern suggest that the revised factor is best understood as rigid conscientious control anchored especially in order/detail orientation and rule-bound responsibility, with perfectionism-related content retained as part of the broader construct.

The original Perfectionism (C1) facet warrants particular consideration. The results do not suggest that perfectionism-related content was irrelevant to the Swedish FFOCI-SF. Rather, Perfectionism (C1) showed limited support as an independent four-item facet. Its internal consistency was low, two Perfectionism (C1) items were removed because of weak and unstable loadings, and the two retained Perfectionism (C1) items were absorbed into the broader C1–C3 factor. Inspection of item content suggested that the removed Perfectionism (C1) items emphasized achievement, efficiency, or pride in work quality, content that may reflect relatively adaptive forms of conscientiousness rather than the rigid, error-focused, and self-critical perfectionism typically associated with OCPD [1, 31]. This interpretation parallels distinctions in the perfectionism literature between relatively adaptive, low-neuroticism strivings and more distress-linked, high-neuroticism perfectionistic concerns [38]. Alternatively, translation- or culture-specific nuances may have attenuated the maladaptive meaning of these items in Swedish respondents.

A related interpretive issue concerns the revised Doggedness (C5) facet. Doggedness (C5) remains theoretically relevant to the FFOCI-SF framework as an indicator of rigid persistence and excessive determination. However, the revised facet contains only two items, limiting content coverage and precluding conventional internal consistency estimation. The retained items appear to preserve a narrower maladaptive persistence theme, whereas the removed items reflected partly redundant task-completion content or broader self-discipline content that overlapped empirically with neighboring conscientiousness facets. Accordingly, findings involving Doggedness (C5) should be considered provisional pending replication.

Across most facets, latent correlations indicated clear convergent validity with theoretically corresponding NEO-PI-3 facets (|r| ≈ 0.50–0.97), supporting the Swedish FFOCI-SF’s grounding in the five-factor model. Associations with the self-report OCPD indicator (DIP-Q) also supported OCPD-related convergent validity overall (|r| ≈ 0.21–0.82), with the weakest associations observed for Constricted (O3) and Detached Coldness (E1). Taken together, these findings support the interpretation of the Swedish FFOCI-SF as a brief, OCPD-oriented measure. At the same time, the very high associations observed for some corresponding NEO-PI-3 facet pairs underscore that convergence should not be equated with incremental validity. Because the present study did not test whether the FFOCI-SF predicts clinically relevant outcomes beyond corresponding NEO-PI-3 facets, such incremental value remains an empirical question. The comparatively weaker DIP-Q associations for Constricted (O3) and Detached Coldness (E1) are compatible with their intended role as facets indexing interpersonal–affective restriction, a content area that only partially overlaps with the control- and perfectionism-related features emphasized in traditional OCPD criteria [36]. Consistent with this distinction, they showed limited associations with the conscientiousness-related facets and Excessive Worry (N1), while correlating strongly with each other. Similar patterns have been reported previously for both the FFOCI-SF and the full FFOCI [18, 36]. At the same time, both facets demonstrated acceptable reliability and a coherent nomological pattern, with strong convergence with theoretically related NEO-PI-3 facets and weak associations with unrelated domains, consistent with their construct validity within the FFOCI-SF framework as interpersonal–affective facets.

The high observed correlation between Risk Aversion (E5) and Inflexibility (O4) also warrants comment, particularly because both facets were retained as separate factors in the revised SEM solution. Inspection of the original facet definitions and retained item content suggests that their association is understandable, as both facets involve preference for predictable and familiar options [36]. However, this overlap does not by itself establish redundancy. Risk Aversion (E5) centers on cautious decision making, including reluctance to take chances, playing it safe, and preferring safe or secure options over exciting or dangerous ones. Inflexibility (O4) centers on rigidity in routines and behavioral patterns, including preference for schedules, tried-and-true approaches, and predictability over exploring alternatives. Thus, despite their high observed intercorrelation in the present sample, the facets can be interpreted as conceptually distinct but closely related expressions of OCPD-relevant rigidity: cautious risk avoidance versus rigid adherence to familiar routines.

Although full measurement invariance was not supported, multiple indicators of psychometric functioning, including reliability, intercorrelations, and associations with external criteria, were broadly similar across the eating disorder and comparison groups. However, given the lack of full invariance support, the two-item Doggedness (C5) facet, the EDE-Q-based group definition, and the elevated psychiatric morbidity in the comparison group, these findings should be interpreted as preliminary known-groups evidence rather than definitive clinical validation. The pattern of group differences was consistent across both latent and observed-score analyses. The eating disorder group reported higher scores on nearly all facets, with the largest difference observed for Excessive Worry (N1) and moderate effects across seven additional facets. In contrast, Constricted (O3) and Detached Coldness (E1) showed the smallest effects, with Constricted (O3) showing no significant group difference, consistent with the validity patterns discussed above. This may appear counterintuitive given that interpersonal difficulties are common in eating disorders [2, 40]. However, these difficulties are typically characterized by heightened social threat sensitivity, anxiety, and avoidance rather than by interpersonal coldness or emotional detachment [25, 40], and recent work on OCPD specifically suggests that social interaction anxiety may play a more prominent role than traditionally emphasized [37]. This threat-sensitive interpersonal inhibition only partially overlaps with the interpersonal–affective restriction indexed by Constricted (O3) and Detached Coldness (E1), which reflect low emotional attunement rather than anxiety-based avoidance [36]. The comparatively weaker group differences for Constricted (O3) and Detached Coldness (E1) thus likely reflect limited construct overlap with the predominant interpersonal phenotype in eating disorders rather than an absence of interpersonal impairment per se. The absence of a facet capturing this anxiety-driven interpersonal pathway highlights a potential gap in the current FFOCI-SF framework, particularly given evidence that individuals with OCPD may differ substantially across interpersonal profiles [31]. Because this study did not include direct measures of interpersonal fear or avoidance, these distinctions warrant further investigation. Importantly, however, this observation concerns the boundaries of the instrument’s construct coverage rather than its psychometric integrity within its intended scope.

### Limitations

This study has several limitations. First, all data were obtained through online, self-administered questionnaires, and to mitigate inattentive responding, embedded attention check items and forced-response prompts were used. Second, measurement invariance analyses did not support configural, metric, or scalar invariance across groups. Given the complexity of the 10-factor model, unequal group sizes, and few items per facet, these analyses should be regarded as exploratory, and latent mean differences interpreted cautiously. Third, sample characteristics constrain generalizability. Participants were predominantly women (88%), highly educated (57% with ≥ 3 years of university studies), and nearly half reported at least one psychiatric diagnosis, indicating a self-selected sample with elevated symptoms relative to population estimates [27, 32]. Notably, approximately one-third of the non-clinical group also reported a recent psychiatric diagnosis, and group comparisons should therefore be interpreted in light of generally elevated psychopathology, which may limit generalizability to truly population-based non-clinical samples. Fourth, eating disorder group membership was defined using an EDE-Q cutoff rather than diagnostic verification. Some individuals with anorexia nervosa may underreport symptoms on the EDE-Q [41], introducing a risk of misclassification. However, self-reported anorexia nervosa diagnoses were substantially more common in the eating disorder group, reducing concerns about systematic misclassification. Taken together, these issues mean that group differences should be interpreted as preliminary known-groups evidence rather than validation against diagnostically verified eating disorder and healthy control groups. Fifth, several items were removed due to weak loadings, cross-loadings, or redundancy, and C1–C3 were consolidated into a single broader facet, limiting direct comparability with the original FFOCI-SF. Nevertheless, the revised structure retained coherent intercorrelations and expected validity patterns, suggesting improved structural clarity without loss of construct coverage. Sixth, the NEO-PI-3 Openness to Values facet showed low internal consistency (α = 0.50), consistent with previous Swedish validations [23]. Because this facet served as the convergent comparator for Dogmatism (O6), the observed association is likely attenuated by measurement error. Finally, Doggedness (C5) was reduced to two items. Although two-item facets are not uncommon in short-form instruments, this limits content coverage, precludes robust internal consistency estimation, and prevented latent group comparisons for this facet.

### Implications for future research

Given the revised facet structure, future research should examine the test–retest reliability and sensitivity to change of the FFOCI-SF, particularly in longitudinal and treatment studies involving individuals with elevated eating disorder or OCPD traits. Replication in more diverse Swedish samples, including populations with lower overall psychopathology, would further strengthen generalizability. Future studies should also examine criterion-related and incremental validity against additional indicators of psychopathology commonly associated with OCPD, including whether FFOCI-SF facets add predictive value beyond corresponding NEO-PI-3 facets for eating disorder symptoms, anxiety, depression, impairment, or other OCPD-relevant outcomes. Future studies should also evaluate whether additional or alternative items from the full FFOCI item pool could improve coverage of facets affected by item removal or merging, particularly Doggedness (C5) and the revised C1–C3 factor. Finally, given the distinct patterns observed for Constricted (O3) and Detached Coldness (E1), further research should examine their facet-specific criterion sensitivity across different assessment contexts to clarify their contribution within the broader FFOCI-SF framework.

## Conclusions

This study provides evidence for a revised 10-factor, 41-item Swedish FFOCI-SF with clear facet delineation, acceptable-to-excellent reliability, and coherent convergent and discriminant validity. Several indicators suggested broadly comparable psychometric functioning across adults with and without elevated eating disorder symptoms, with systematically higher OCPD trait levels observed in the eating disorder group across most facets. These findings support further use and evaluation of the Swedish FFOCI-SF as a dimensional measure of OCPD traits in both research and clinical contexts, including studies of adults with elevated eating disorder symptoms.

## Electronic Supplementary Material

Below is the link to the electronic supplementary material.

Supplementary Material 1

## Data Availability

The datasets used and/or analysed during the current study are available from the corresponding author on reasonable request.

## Abbreviations

FFOCI-SF: Five Factor Obsessive-Compulsive Inventory–Short Form
OCPD: Obsessive-compulsive personality disorder
EDE-Q: Eating Disorder Examination Questionnaire
NEO-PI-3: NEO Personality Inventory-3
DIP-Q: DSM-IV and ICD-10 Personality Questionnaire
CFA: Confirmatory factor analysis
SEM: structural equation modeling
CTT: Classical test theory
CITC: Corrected item–total correlation
RMSEA: Root mean square error of approximation
CFI: Comparative fit index
SRMR: Standardized root mean square residual
MLR: Robust maximum likelihood
