# Supplementary material for: Adaptation and validation of the Swedish Five Factor Obsessive-Compulsive Inventory-Short Form in adults with and without eating disorder psychopathology
Source: BMC Psychiatry. 2026 Jul 15;26:538. doi: 10.1186/s12888-026-08355-9 (PMC13374307; doi:10.1186/s12888-026-08355-9)
Supplement: Supplementary file 1 — Supplementary Material 1 [file 12888_2026_8355_MOESM1_ESM.docx]

**Supplementary Table 1**. Item Wording, Translation, and Revised Scoring of the Swedish FFOCI-SF

| **Item number** | **Original English item** | **Swedish translated item** | **Original subscale assignment** | **Revised subscale assignment** | **Status** |
| --- | --- | --- | --- | --- | --- |
| 1 | I often worry about the future | Jag oroar mig ofta för framtiden | N1 Excessive Worry | N1 Excessive Worry | Retained |
| 2 | I am a warm and engaging person | Jag är en varm och engagerad person | E1 Detached Coldness | E1 Detached Coldness | Retained |
| 3 | I love the excitement of making risky decisions | Jag älskar spänningen i att ta riskfyllda beslut | E5 Risk Aversion | E5 Risk Aversion | Retained |
| 4 | I am not a person who is into how people feel about things | Jag är inte så intresserad av hur andra människor känner inför saker och ting | O3 Constricted | O3 Constricted | Retained |
| 5 | My life is on such a schedule that others do find me a bit dull at times | Mitt liv är schemalagt till den grad att andra nog tycker att jag är lite tråkig ibland | O4 Inflexibility | O4 Inflexibility | Retained |
| 6 | I live my life by a set of tough, unyielding moral principles | Jag lever mitt liv efter strikta och orubbliga moraliska principer | O6 Dogmatism | O6 Dogmatism | Retained |
| 7 | I take great pride in the quality of my work | Jag är väldigt stolt över kvaliteten på det arbete jag utför | C1 Perfectionism | Not retained in revised scoring | Removed |
| 8 | I need to consider every little detail | Jag måste överväga varje liten detalj | C2 Fastidiousness | Not retained in revised scoring | Removed |
| 9 | I firmly believe that you should always play strictly by the rules | Jag är övertygad om att man alltid ska hålla sig strikt till de regler som finns | C3 Punctiliousness | Not retained in revised scoring | Removed |
| 10 | I get so caught up in my work that I lose time for other things. | Jag fastnar så mycket i mitt arbete att jag knappt har tid till annat | C4 Workaholism | C4 Workaholism | Retained |
| 11 | I'm fanatical about getting things done when they need to be. | Jag är verkligen noga med att få gjort saker i tid | C5 Doggedness | C5 Doggedness | Retained |
| 12 | No decision is too small for me not to think through all the consequences. | Jag tänker noga igenom konsekvenserna av alla beslut jag tar | C6 Ruminative Deliberation | C6 Ruminative Deliberation | Retained |
| 13 | I ruminate and worry over lots of different things | Jag grubblar och oroar mig för en mängd olika saker | N1 Excessive Worry | N1 Excessive Worry | Retained |
| 14 | I enjoy getting to know people on a personal level | Jag tycker om att lära känna människor på ett personligt plan | E1 Detached Coldness | E1 Detached Coldness | Retained |
| 15 | I much prefer playing it safe, even if miss out on something | Jag föredrar att vara försiktig även om jag går miste om saker i livet på grund av det | E5 Risk Aversion | E5 Risk Aversion | Retained |
| 16 | I find it difficult to feel what other people are feeling | Jag har svårt att känna vad andra känner | O3 Constricted | O3 Constricted | Retained |
| 17 | I like to keep to the “tried and true” rather than try new things | Jag håller mig till det jag vet fungerar snarare än att pröva nya saker | O4 Inflexibility | O4 Inflexibility | Retained |
| 18 | There is never an excuse for deviating from a moral code | Det är aldrig okej att avvika från det som anses riktigt | O6 Dogmatism | O6 Dogmatism | Retained |
| 19 | I'm something of a perfectionist | Jag är något av en perfektionist | C1 Perfectionism | C1-C3 combined facet: rigid conscientious control | Retained |
| 20 | Other people have said that I'm extremely detail oriented, almost to a fault | Andra har sagt att jag är överdrivet, nästan extremt, detaljorienterad | C2 Fastidiousness | C1-C3 combined facet: rigid conscientious control | Retained |
| 21 | Following the rules is always important, even if I'm playing a game by myself | Att följa reglerna är alltid viktigt, även om jag spelar ett spel i ensamhet | C3 Punctiliousness | C1-C3 combined facet: rigid conscientious control | Retained |
| 22 | I am known as something of a "workaholic." | Andra kan beskriva mig som en arbetsnarkoman | C4 Workaholism | C4 Workaholism | Retained |
| 23 | If I start something I work until it is complete | När jag påbörjar något slutför jag det alltid | C5 Doggedness | Not retained in revised scoring | Removed |
| 24 | I examine every detail of an issue before coming to a decision | Jag undersöker varje detalj innan jag fattar ett beslut | C6 Ruminative Deliberation | C6 Ruminative Deliberation | Retained |
| 25 | I am a worrier | Jag oroar mig mycket | N1 Excessive Worry | N1 Excessive Worry | Retained |
| 26 | I must admit that I am not a particularly warm person | Jag måste erkänna att jag inte är en särskilt varm person | E1 Detached Coldness | E1 Detached Coldness | Retained |
| 27 | I believe that safe and predictable beats exciting and dangerous every time | Jag tror att det säkra och förutsägbara alternativet är bättre än det spännande och riskfyllda i alla lägen | E5 Risk Aversion | E5 Risk Aversion | Retained |
| 28 | Strong emotions are not that important in my life | Starka känslor är inte så viktiga i mitt liv | O3 Constricted | O3 Constricted | Retained |
| 29 | My life is pretty much the same every week, and that's how I like it | Mitt liv är ungefär detsamma varje vecka och det är så jag gillar det | O4 Inflexibility | O4 Inflexibility | Retained |
| 30 | Permissiveness is essentially a break down in morality | Att vara tolerant och överseende är detsamma som moraliskt förfall | O6 Dogmatism | O6 Dogmatism | Retained |
| 31 | I take great pride in being efficient and effective | Jag är stolt över att vara effektiv | C1 Perfectionism | Not retained in revised scoring | Removed |
| 32 | I probably spend more time than is needed organizing and ordering things | Jag lägger nog mer tid än vad som är nödvändigt på att ordna och organisera saker och ting | C2 Fastidiousness | C1-C3 combined facet: rigid conscientious control | Retained |
| 33 | People often suggest I take my responsibilities too seriously | Folk antyder ofta att jag tar mina plikter på för stort allvar | C3 Punctiliousness | C1-C3 combined facet: rigid conscientious control | Retained |
| 34 | I don't regret working real hard because, for me, work is my pleasure | Jag ångrar inte att jag har jobbat riktigt hårt eftersom arbete är det jag finner nöje i | C4 Workaholism | C4 Workaholism | Retained |
| 35 | I have an extremely strong sense of self-discipline | Jag har mycket stark självdisciplin | C5 Doggedness | Not retained in revised scoring | Removed |
| 36 | I like to be really sure about things before I act | Jag gillar att vara riktigt säker innan jag agerar | C6 Ruminative Deliberation | C6 Ruminative Deliberation | Retained |
| 37 | I am often concerned, even nervous, about things going wrong | Jag blir ofta orolig, till och med nervös, över saker som kan gå fel | N1 Excessive Worry | N1 Excessive Worry | Retained |
| 38 | Warmth and intimacy are not my strengths | Värme och intimitet hör inte till mina styrkor | E1 Detached Coldness | E1 Detached Coldness | Retained |
| 39 | My way of life might be dull to others, but at least it is safe and secure | Mitt sätt att leva kan säkert uppfattas som tråkigt, men det är åtminstone tryggt och säkert | E5 Risk Aversion | E5 Risk Aversion | Retained |
| 40 | I don't experience a particularly wide range of emotions or feelings | Jag upplever inte ett särskilt brett spektrum av känslor | O3 Constricted | O3 Constricted | Retained |
| 41 | I much prefer predictability than exploring the unknown | Jag föredrar förutsägbarhet snarare än att utforska det okända | O4 Inflexibility | O4 Inflexibility | Retained |
| 42 | I don't believe in excuses for violating an ethical, moral code | Inga ursäkter finns för att bryta mot det som är etiskt och moraliskt riktigt | O6 Dogmatism | O6 Dogmatism | Retained |
| 43 | I like my work to be flawless and unblemished | Jag gillar när mitt arbete är felfritt | C1 Perfectionism | C1-C3 combined facet: rigid conscientious control | Retained |
| 44 | I always make sure that my work is very well planned and organized | Jag ser alltid till att mitt arbete är mycket välplanerat och organiserat | C2 Fastidiousness | C1-C3 combined facet: rigid conscientious control | Retained |
| 45 | Some persons suggest I can be excessive in my emphasis on being proper and moral | Några menar att jag kan vara överdrivet ordentlig och moralisk | C3 Punctiliousness | C1-C3 combined facet: rigid conscientious control | Retained |
| 46 | While others are playing, I'm getting ahead | Medan andra roar sig i stunden planerar jag inför framtiden | C4 Workaholism | Not retained in revised scoring | Removed |
| 47 | I am to the maximum dogged, determined, and disciplined | Jag är envis och disciplinerad till max | C5 Doggedness | C5 Doggedness | Retained |
| 48 | I think things over and over and over before I make a decision | Jag tänker igenom saker om och om och om igen innan jag fattar ett beslut | C6 Ruminative Deliberation | C6 Ruminative Deliberation | Retained |

*Note*. Original subscale assignments follow the English FFOCI-SF scoring algorithm. The revised subscale assignment reflects the final 41-item scoring used in the present study. Gray shading indicates removed items. FFOCI-SF copyright © 2014 Douglas B. Samuel, Ashley D. B. Riddell, Donald R. Lynam, Joshua D. Miller, and Thomas A. Widiger.

**Supplementary Table 2.** Original 48-item FFOCI-SF scoring algorithm

| **Subscale** | **Scoring algorithm** |
| --- | --- |
| N1 | Excessive Worry: 1 + 13 + 25 + 37 |
| E1 | Detached Coldness: 2 (R) + 14 (R) + 26 + 38 |
| E5 | Risk Aversion: 3 (R) + 15 + 27 + 39 |
| O3 | Constricted: 4 + 16 + 28 + 40 |
| O4 | Inflexibility: 5 + 17 + 29 + 41 |
| O6 | Dogmatism: 6 + 18 + 30 + 42 |
| C1 | Perfectionism: 7 + 19 + 31 + 43 |
| C2 | Fastidiousness: 8 + 20 + 32 + 44 |
| C3 | Punctiliousness: 9 + 21 + 33 + 45 |
| C4 | Workaholism: 10 + 22 + 34 + 46 |
| C5 | Doggedness: 11 + 23 + 35 + 47 |
| C6 | Ruminative Deliberation: 12 + 24 + 36 + 48 |

*Note*. R = reverse-scored item. FFOCI-SF = Five-Factor Obsessive–Compulsive Inventory–Short Form

## **Supplementary Table 3.** Revised 41-item scoring algorithm used in the present study

| **Subscale** | **Scoring algorithm** |
| --- | --- |
| N1 | Excessive Worry: 1 + 13 + 25 + 37 |
| E1 | Detached Coldness: 2 (R) + 14 (R) + 26 + 38 |
| E5 | Risk Aversion: 3 (R) + 15 + 27 + 39 |
| O3 | Constricted: 4 + 16 + 28 + 40 |
| O4 | Inflexibility: 5 + 17 + 29 + 41 |
| O6 | Dogmatism: 6 + 18 + 30 + 42 |
| C1-C3† | Rigid Conscientious Control: 19 + 20 + 21 + 32 + 33 + 43 + 44 + 45 |
| C4† | Workaholism: 10 + 22 + 34 |
| C5† | Doggedness: 11 + 47 |
| C6 | Ruminative Deliberation: 12 + 24 + 36 + 48 |

*Note*. The revised scoring algorithm is specific to the Swedish validation analyses reported in the present study and should not be interpreted as replacing the original FFOCI-SF scoring algorithm without further validation. †Subscale scoring differs from the original FFOCI-SF scoring algorithm. R = reverse-scored item. FFOCI-SF = Five-Factor Obsessive-Compulsive Inventory–Short Form.

**Supplementary Table 4.** Item-Level Psychometric Properties for the Original FFOCI-SF

| Subscale | Item | *M* | *SD* | Skewness | Kurtosis | CITC (subscale) | CITC (full scale) | α (total sample) | α if item deleted (subscale) | α (non-clinical) | α (ED) | Inter-item r, M (range) |
| --- | --- | --- | --- | --- | --- | --- | --- | --- | --- | --- | --- | --- |
| N1 | Item 1 | 3.48 | 1.16 | -0.40 | -0.73 | .74 | .36 | .92 | .92 | .93 | .88 | .75 (.62–.87) |
|  | Item 13 | 3.62 | 1.28 | -0.61 | -0.74 | .88 | .48 |  | .88 |  |  |  |
|  | Item 25 | 3.56 | 1.30 | -0.48 | -0.90 | .88 | .49 |  | .88 |  |  |  |
|  | Item 37 | 3.58 | 1.28 | -0.51 | -0.91 | .78 | .51 |  | .91 |  |  |  |
| E1 | Item 2 | 2.04 | 0.83 | 0.55 | 0.01 | .59 | .12 | .78 | .73 | .79 | .76 | .48 (.37–.63) |
|  | Item 14 | 1.93 | 0.98 | 0.93 | 0.32 | .46 | .22 |  | .78 |  |  |  |
|  | Item 26 | 1.87 | 0.95 | 0.89 | 0.07 | .70 | .27 |  | .66 |  |  |  |
|  | Item 38 | 2.18 | 1.24 | 0.83 | -0.37 | .63 | .33 |  | .71 |  |  |  |
| E5 | Item 3 | 3.80 | 1.01 | -0.60 | -0.33 | .37 | .10 | .74 | .76 | .73 | .73 | .41 (.22–.57) |
|  | Item 15 | 2.99 | 1.05 | 0.11 | -0.64 | .64 | .53 |  | .61 |  |  |  |
|  | Item 27 | 2.93 | 1.02 | -0.09 | -0.56 | .57 | .53 |  | .65 |  |  |  |
|  | Item 39 | 2.89 | 1.19 | 0.02 | -0.85 | .54 | .52 |  | .67 |  |  |  |
| O3 | Item 4 | 1.94 | 1.00 | 1.01 | 0.50 | .33 | .07 | .70 | .72 | .68 | .72 | .37 (.19–.57) |
|  | Item 16 | 1.81 | 1.01 | 1.25 | 0.95 | .53 | .22 |  | .61 |  |  |  |
|  | Item 28 | 2.06 | 1.05 | 0.88 | 0.16 | .52 | .16 |  | .61 |  |  |  |
|  | Item 40 | 1.86 | 1.10 | 1.16 | 0.35 | .56 | .28 |  | .58 |  |  |  |
| O4 | Item 5 | 2.52 | 1.16 | 0.29 | -0.80 | .46 | .57 | .77 | .78 | .75 | .77 | .46 (.34–.65) |
|  | Item 17 | 2.96 | 1.01 | 0.01 | -0.52 | .60 | .53 |  | .70 |  |  |  |
|  | Item 29 | 2.96 | 1.04 | -0.03 | -0.57 | .55 | .41 |  | .72 |  |  |  |
|  | Item 41 | 3.02 | 1.08 | 0.00 | -0.73 | .67 | .57 |  | .66 |  |  |  |
| O6 | Item 6 | 2.62 | 1.09 | 0.10 | -0.84 | .39 | .45 | .71 | .67 | .65 | .73 | .38 (.23–.53) |
|  | Item 18 | 2.56 | 1.06 | 0.15 | -0.79 | .36 | .41 |  | .58 |  |  |  |
|  | Item 30 | 1.75 | 0.83 | 0.96 | 0.61 | .40 | .47 |  | .71 |  |  |  |
|  | Item 42 | 2.63 | 1.18 | 0.17 | -0.96 | .50 | .42 |  | .60 |  |  |  |
| C1 | Item 7† | 3.46 | 1.00 | -0.53 | -0.12 | .20 | -.02 | .54 | .58 | .53 | .60 | .24 (.07–.47) |
|  | Item 19 | 3.21 | 1.16 | -0.32 | -0.70 | .32 | .55 |  | .49 |  |  |  |
|  | Item 31† | 3.86 | 1.00 | -0.77 | 0.06 | .35 | .28 |  | .46 |  |  |  |
|  | Item 43 | 4.21 | 0.86 | -1.03 | 0.82 | .49 | .48 |  | .36 |  |  |  |
| C2 | Item 8† | 2.86 | 1.17 | 0.18 | -0.87 | .52 | .58 | .77 | .74 | .76 | .74 | .45 (.36–.53) |
|  | Item 20 | 2.43 | 1.21 | 0.43 | -0.80 | .61 | .63 |  | .68 |  |  |  |
|  | Item 32 | 2.82 | 1.29 | 0.14 | -1.10 | .59 | .56 |  | .70 |  |  |  |
|  | Item 44 | 3.43 | 1.08 | -0.38 | -0.39 | .55 | .64 |  | .72 |  |  |  |
| C3 | Item 9† | 2.72 | 1.01 | -0.04 | -0.84 | .53 | .56 | .70 | .61 | .68 | .68 | .37 (.22–.55) |
|  | Item 21 | 2.69 | 1.26 | 0.18 | -1.10 | .45 | .43 |  | .66 |  |  |  |
|  | Item 33 | 2.81 | 1.21 | 0.04 | -1.01 | .45 | .58 |  | .66 |  |  |  |
|  | Item 45 | 2.81 | 1.13 | -0.03 | -0.89 | .52 | .52 |  | .62 |  |  |  |
| C4 | Item 10 | 2.49 | 1.01 | 0.33 | -0.64 | .55 | .44 | .75 | .70 | .72 | .79 | .43 (.35–.57) |
|  | Item 22 | 2.54 | 1.22 | 0.41 | -0.76 | .65 | .37 |  | .64 |  |  |  |
|  | Item 34 | 2.96 | 1.21 | -0.07 | -0.93 | .55 | .32 |  | .69 |  |  |  |
|  | Item 46† | 2.84 | 1.14 | 0.07 | -0.76 | .46 | .61 |  | .75 |  |  |  |
| C5 | Item 11 | 3.68 | 1.12 | -0.66 | -0.28 | .55 | .45 | .78 | .75 | .80 | .76 | .47 (.35–.62) |
|  | Item 23† | 3.20 | 1.17 | -0.28 | -0.78 | .59 | .29 |  | .73 |  |  |  |
|  | Item 35† | 3.25 | 1.22 | -0.25 | -0.79 | .63 | .37 |  | .70 |  |  |  |
|  | Item 47 | 2.90 | 1.24 | 0.00 | -1.02 | .57 | .55 |  | .73 |  |  |  |
| C6 | Item 12 | 2.98 | 1.13 | 0.01 | -1.12 | .70 | .62 | .88 | .87 | .86 | .90 | .66 (.55–.71) |
|  | Item 24 | 2.94 | 1.16 | 0.15 | -0.85 | .80 | .65 |  | .83 |  |  |  |
|  | Item 36 | 3.45 | 1.05 | -0.38 | -0.46 | .72 | .60 |  | .86 |  |  |  |
|  | Item 48 | 3.34 | 1.16 | -0.26 | -0.71 | .77 | .62 |  | .84 |  |  |  |

*Note. n* = 395 (non-clinical = 243; eating disorder = 152). ED = eating disorder group. FFOCI-SF = Five-Factor Obsessive–Compulsive Inventory–Short Form. Subscale abbreviations: N1 = Excessive Worry; E1 = Detached Coldness; E5 = Risk Aversion; O3 = Constricted; O4 = Inflexibility; O6 = Dogmatism; C1 = Perfectionism; C2 = Fastidiousness; C3 = Punctiliousness; C4 = Workaholism; C5 = Doggedness; C6 = Ruminative Deliberation. CITC (subscale) = corrected item–total correlation with its own subscale (item removed). CITC (full scale) = corrected item–total correlation with the original 48-item total score (item removed); α if deleted = Cronbach’s α for the subscale if the given item is removed. Inter-item r represents mean within-subscale Pearson correlations (range in parentheses). † = Item removed in the final version.

**Supplementary Table 5.** Psychometric Properties of Revised FFOCI-SF Subscales

| Subscale | Item | CITC (subscale) | CITC (full scale) | α (total sample) | α if item deleted (subscale) | α (non-clinical) | α (ED) | Inter-item r, M (range) |
| --- | --- | --- | --- | --- | --- | --- | --- | --- |
| C1-C3 | 19 | .61 | .52 | .84 | .81 | .83 | .83 | .39 (.22–.62) |
|  | 20 | .67 | .62 | – | .80 | – | – | – |
|  | 21 | .36 | .43 | – | .85 | – | – | – |
|  | 32 | .63 | .53 | – | .81 | – | – | – |
|  | 33 | .61 | .55 | – | .81 | – | – | – |
|  | 43 | .51 | .45 | – | .82 | – | – | – |
|  | 44 | .60 | .60 | – | .81 | – | – | – |
|  | 45 | .57 | .52 | – | .82 | – | – | – |
| C4 | 10 | .54 | .41 | .75 | .70 | .71 | .77 | .49 (.37–.57) |
|  | 22 | .67 | .33 | – | .54 | – | – | – |
|  | 34 | .52 | .26 | – | .73 | – | – | – |
| C5 | 11 | N/A | .38 | N/A | N/A | N/A | N/A | .35 |
|  | 47 | N/A | .49 | – | N/A | – | – | – |

*Note. n* = 395 (non-clinical = 243; ED = 152). ED = eating disorder group. Revised FFOCI-SF = 41-item version derived from the final SEM model. FFOCI-SF = Five-Factor Obsessive–Compulsive Inventory–Short Form. Subscales are the revised C1-C3 (combined Perfectionism, Fastidiousness, and Punctiliousness, interpreted as rigid conscientious control) revised C4 (Workaholism), and revised C5 (Doggedness). CITC (subscale) = corrected item–total correlation within the subscale (item removed). CITC (scale) = corrected item–total correlation with the revised 41-item total score (item removed). α if deleted = Cronbach’s α for the subscale if the given item is removed. Inter-item r represents mean within-subscale Pearson correlations (range in parentheses). For the two-item Doggedness (C5) facet, internal consistency was not estimated, as coefficient alpha is not informative for two-item scales.

**Supplementary Table 6.** Between-Group Differences in FFOCI-SF Facet Intercorrelations

|  | C1-C3† | C4† | C5† | C6 | E1 | E5 | N1 | O3 | O4 | O6 |
| --- | --- | --- | --- | --- | --- | --- | --- | --- | --- | --- |
| C1-C3† |  | 1.44 | -0.24 | -1.41 | 0.72 | -0.81 | 0.04 | 0.16 | 0.1 | 1.54 |
| C4† |  |  | 1.05 | -0.7 | -0.55 | -0.81 | -0.31 | 0.51 | 0.02 | 0.49 |
| C5† |  |  |  | -0.87 | -0.11 | -1.45 | -0.28 | 0.76 | 1.15 | 0.04 |
| C6 |  |  |  |  | 0.78 | 0.13 | 2.07‡* | 0.09 | 0.66 | 0.63 |
| E1 |  |  |  |  |  | 1.15 | 0.15 | 0.37 | 0.38 | 1.22 |
| E5 |  |  |  |  |  |  | 0.14 | 1.04 | 1.01 | 0.73 |
| N1 |  |  |  |  |  |  |  | 1.14 | 0.04 | 0.80 |
| O3 |  |  |  |  |  |  |  |  | 0.22 | 0.91 |
| O4 |  |  |  |  |  |  |  |  |  | 1.37 |

*Note*. *n* = 395 (non-clinical = 243; eating disorder = 152). Only the lower triangle is reported; cells below the diagonal are intentionally left blank to avoid redundancy. † = Post-SEM revised scales are based on the final item configuration. FFOCI-SF† = Five-Factor Obsessive–Compulsive Inventory–Short Form (total score; 41 items). FFOCI-SF subscales: C1–C3† = combined Perfectionism, Fastidiousness, and Punctiliousness, interpreted as rigid conscientious control; C4† = Workaholism; C5† = Doggedness; C6 = Ruminative Deliberation; E1 = Detached Coldness; E5 = Risk Aversion; N1 = Excessive Worry; O3 = Constricted; O4 = Inflexibility; O6 = Dogmatism. Positive z values indicate stronger correlations in the clinical group; negative z values indicate stronger correlations in the non-clinical group. ‡ = *r* (eating disorder): .593; *r* (non-clinical): .435. p < .05 (*).

**Supplementary Table 7.** Convergent and Discriminant Validity Based on Observed-Score Correlations

|  | C1-C3† | C4† | C5† | C6 | N1 | E1 | E5 | O3 | O4 | O6 |
| --- | --- | --- | --- | --- | --- | --- | --- | --- | --- | --- |
| NEO facet‡ | .31^*^ | .61^*^ | .60^*^ | .61^*^ | .87^*^ | -.71^*^ | -.44^*^ | -.73^*^ | -.75^*^ | -.31^*^ |
| Disc same§ | .41 (.29–.56) | .18 (.02–.32) | .44 (.24–.59) | .24 (.08–.39) | .54 (.12-.73) | -.33 (-.52--.14) | -.38 (-.45- -.35) | -.20 (-.30--.13) | -.25 (-.33-.17) | -.16 (-.36--.03) |
| Disc other¶ | -.05 (-.45–.35) | .02 (-.14–.32) | -.03 (-.34-.22) | -.03 (-.44-.48) | -.11 (-.47-.28) | -.13 (-.61-.29) | .04 (-.65-.51) | -.13 (-.44-.06) | .02 (-.39-.42) | .02 (-.30-.24) |
| DIP-Q OCPD | .58^*^ | .35^*^ | .31^*^ | .53^*^ | .47^*^ | .20^*^ | .38^*^ | .11^*^ | .43^*^ | .41^*^ |

*Note*. *n* = 395. †Revised FFOCI-SF scales reflect the final 41-item configuration supported by the SEM analyses. FFOCI-SF subscales: C1–C3† = combined Perfectionism, Fastidiousness, and Punctiliousness, interpreted as rigid conscientious control; C4† = Workaholism; C5† = Doggedness; C6 = Ruminative Deliberation; E1 = Detached Coldness; E5 = Risk Aversion; N1 = Excessive Worry; O3 = Constricted; O4 = Inflexibility; O6 = Dogmatism. NEO facet‡ = corresponding NEO-PI-3 facet. For C1–C3, the NEO-facet value represents the mean of the three corresponding NEO-PI-3 facets (C1, C2, C3). Disc same§ = discriminant validity calculated as the mean (range) of correlations with noncorresponding NEO-PI-3 facets within the same domain. Disc other¶ = discriminant validity calculated as the mean (range) of correlations with NEO-PI-3 facets outside the subscale’s domain. DIP-Q OCPD = DSM-IV and ICD-10 Personality Questionnaire (10 OCPD items). p < .05 (*).

**Supplementary Table 8.** Between-Group Differences in Convergent and Discriminant Validity

|  | C1-C3† | C4† | C5† | C6 | N1 | E1 | E5 | O3 | O4 | O6 |
| --- | --- | --- | --- | --- | --- | --- | --- | --- | --- | --- |
| NEO facet^‡^ | 0.27 | 0.15 | 0.34 | 1.89 | 0.27 | 0.89 | 0.17 | 0.39 | 1.39 | 0.89 |
| DIP-Q OCPD | 1.38 | 1.37 | 1.26 | 0.2 | 0.38 | 0.33 | 1.45 | 0.49 | 1.35 | 0.31 |

*Note*. *n* = 395 (non-clinical = 243; eating disorder = 152). †Revised FFOCI-SF scales reflect the final 41-item configuration supported by the SEM analyses. FFOCI-SF subscales: C1–C3† = combined Perfectionism, Fastidiousness, and Punctiliousness, interpreted as rigid conscientious control; C4† = Workaholism; C5† = Doggedness; C6 = Ruminative Deliberation; E1 = Detached Coldness; E5 = Risk Aversion; N1 = Excessive Worry; O3 = Constricted; O4 = Inflexibility; O6 = Dogmatism. NEO facet‡ = corresponding Revised NEO-PI-3 facet. For C1–C3†, the NEO facet value represents the mean (range) of the three corresponding NEO-PI-3 facets (C1, C2, C3). DIP-Q OCPD = DSM-IV and ICD-10 Personality Questionnaire (10 OCPD items). p < .05 (*).
